# Diverticular disease of the colon: evolution of the therapeutic approach and the role of computed tomography in the evaluation of acute conditions

**DOI:** 10.1590/0100-3984.2017.50.2e3

**Published:** 2017

**Authors:** Valdair Francisco Muglia

**Affiliations:** 1 Associate Professor, Department of Clinical Medicine, Center for Imaging Sciences and Medical Physics, Faculdade de Medicina de Ribeirão Preto da Universidade de São Paulo (FMRP-USP), Ribeirão Preto, SP, Brazil. E-mail: fmuglia@fmrp.usp.br

Diverticular disease of the colon is an extremely common disease in the Western world, with an estimated incidence ranging from 5% in middle-aged individuals (those in the fourth decade of life) to as high as 60% in those over 80 years of age^(1)^. The condition has been associated with low fiber intake, increased colonic transit time, and increased intraluminal pressure in the colon, leading to the development of diverticula^(2)^.

It is important that the terms "diverticulosis" and "diverticular disease" be defined correctly. Diverticulosis refers to the occurrence of diverticula, with or without symptoms, usually accompanied by changes in the wall of the colon, such as elastin deposition, smooth muscle thickening, shortening of the taenia, and a consequent reduction of the intestinal lumen^(3)^. In contrast, diverticular disease refers to the presence of diverticula accompanied by major symptoms, such as acute diverticulitis or diverticulitis associated with chronic conditions. Acute diverticulitis can be classified as complicated or uncomplicated.

Although clinical symptoms and signs can suggest the possibility of diverticular disease, the clinical profile and physical examination findings have low diagnostic accuracy. In addition, even when the clinical diagnosis is quite suggestive, the extent of the inflammatory process cannot be well characterized on the basis of the clinical findings. Therefore, ancillary tests such as endoscopy and imaging studies play an important role in the management of diverticular disease^(2,4)^. A number of recent studies in the radiology literature of Brazil have demonstrated the importance of computed tomography (CT) in the evaluation of diseases of the colon^(5-11)^.

In this issue of **Radiologia Brasileira**, Naves et al.^(12)^ discuss the role of imaging methods, particularly CT, in the evaluation of acute colonic diverticulitis (ACD). Considered the imaging method of choice in the evaluation of ACD, CT not only allows the diagnosis to be confirmed but also facilitates the distinction between the complicated and uncomplicated forms. Since 1978, complicated ACD has been categorized according to the system devised by Hinchey et al.^(13)^. However, the Hinchey classification system has some significant limitations. For example, Hinchey stage III ACD can be differentiated from Hinchey stage IV ACD only by laparoscopy or, if appropriate, laparotomy. Other attempts to stratify ACD have recently emerged^(4)^, a theme that is also addressed by the authors^(12)^.

In addition to the discussion of the main complications of ACD, which are well illustrated in their paper, Naves et al.^(12)^ also focus on an extremely relevant issue-the differential diagnosis between ACD and colorectal neoplasia, both of which are relatively common, and therefore often coexist, in elderly patients^(14,15)^. The differentiation between ACD and colorectal neoplasia becomes particularly difficult when diverticular disease presents with significant intestinal wall thickening and a significant reduction in the size of the lumen. The coexistence of those two conditions led several groups of authors to recommend colonoscopy after the resolution of an episode of acute diverticulitis, as typically confirmed by CT^(16)^. However, more recently, a number of other studies, including one meta-analysis, have suggested that the available data do not support that recommendation^(17)^.

Previously, complicated CAD was, in most cases, an indication for a surgical treatment^(1,18)^. However, the indications for surgery have recently become more restrictive and new algorithms from international institutions recommend more conservative options, surgery being reserved for acute cases that require more incisive measures^(19)^. In such approaches, the definition of the extent of the inflammatory process is crucial, requiring a CT examination dedicated to that purpose, with a specific protocol. Another contribution of CT to this new therapeutic approach to ACD is that infected collections secondary to diverticulitis no longer constitute an absolute indication for surgery, such collections now, if possible, being dealt with by CT- or ultrasound-guided percutaneous drainage.

The therapeutic management of ACD, an extremely prevalent condition, has been the subject of ongoing discussions, with major paradigm shifts in recent years^(20)^. However, in all of the suggested options, CT plays a central role as an important means of diagnosis and determination of the extent of the inflammatory process. Therefore, the radiologist plays a well-defined and important role in the multidisciplinary approach to ACD.
